# A multi‐institution performance assessment of a double‐layer MLC

**DOI:** 10.1002/acm2.70261

**Published:** 2025-10-30

**Authors:** Andy Buckle, Conor Heeney, Matthew Jones, Vasu Ganesan, Ronan Valentine, Philip Wheeler, James Earley, Garry Grogan, Matthew Sparks

**Affiliations:** ^1^ Oxford University Hospitals NHS Foundation Trust Radiotherapy Physics Churchill Hospital Oxford UK; ^2^ Mid‐Western Radiation Oncology Centre, Mater Private Network, Mid‐Western Radiation Oncology Centre University Hospital Limerick Dooradoyle Limerick Ireland; ^3^ Royal Surrey County Hospital NHS Foundation Trust Royal Surrey County Hospital Guildford UK; ^4^ Barking, Havering and Redbridge University Hospital NHS Trust Queen's Hospital Romford UK; ^5^ The Beatson West of Scotland Cancer Centre Gartnavel General Hospital Glasgow UK; ^6^ Velindre University NHS Trust Velindre Cancer Centre Cardiff UK; ^7^ Royal Surrey County Hospital NHS Foundation Trust Royal Surrey Cancer Centre Guildford UK; ^8^ University Hospitals Sussex NHS Foundation Trust Sussex Cancer Centre Brighton UK

**Keywords:** audit, Halcyon, MLC, QA

## Abstract

**Background:**

Varian Halcyon and Ethos linacs use a novel double‐layer MLC.

**Purpose:**

To perform a multi‐institutional characterization of MLC performance of Varian Halcyon and Ethos linacs and establish the range of typical performance indices.

**Methods:**

The tests used plans delivered to the megavoltage (MV) imaging panel that can be easily implemented and reproducibly delivered in different centers. Automated image analysis was used to obtain metrics that characterize the MLC performance. The tests included picket fence, MLC leakage, sweeping gap, open field symmetry changes, change of output with gantry angle, and volumetric modulated arc therapy (VMAT) indices. Sixteen linacs from 11 centers were included in the study.

**Results:**

Performance indices were found that are in line with other linacs, with the exception of proximal layer positional calibration.

**Conclusions:**

Typical ranges have been established for Halcyon/Ethos MLC performance indices.

## 1 INTRODUCTION

The Multi Leaf Collimators (MLCs) on Varian Halcyon^1^ linear accelerator have a significant difference in construction compared to the traditional Millenium/HD MLC design on the TrueBeams. Whereas the Millenium MLC on the TrueBeam is a single layer tertiary MLC, the Halcyon MLCs dual layer provides the beam shaping.^2^ The interleaf junction in each layer is in line with the middle of a leaf in the other layer, minimizing interleaf leakage. The Halcyon MLC has a maximum leaf speed of**1** 5.0 cm/s compared to^3^ 2.5 cm/s on the TrueBeam's Millenium 120 MLC.

Evaluating and baselining MLC characteristics is critical both during commissioning and for routine quality assurance (QA) as it enables suboptimal performance to be identified and rectified. A common and effective approach for routine testing of MLC is through utilization of the digital megavoltage imager (DMI).

LoSasso et al.^4^ discussed the potential of DMIs for MLC QA in 2001. Since then, developments in resolution and software have resulted in significant improvement. In 2012 Richart et al.^5^ published their use of a DMI to investigate MLC performance on a single linac. They included picket fence and sweeping gap. They concluded that the method was time efficient, had good coverage of MLC positioning/speed, and gave confidence in delivery. Isono et al.^6^ surveyed a variety of Varian linacs with traditional single layer MLCs using a variety of measuring devices. They focused on parameters required for treatment planning system configuration such as dosimetric leaf gap and transmission.

The aim of this multicenter study was to establish, through the analysis of the images produced by MLC deliveries to the DMI, a set of typical performance indices for quality control of the double layer MLC.

## 2 METHODS

DICOM test plans were created for delivery to the DMI. The test plans were heavily edited versions of the test plans that Varian supply with the linac. Most of the plans contained open fields, which were used to normalize the test field images removing the flattening filter‐free (FFF) peak. The majority of the plans tested both the proximal and distal MLC layers separately.

The plans were delivered on 16 linacs across 11 centers. The Halcyon/Ethos platform may be configured to a choice of reference calibration conditions. Table 1 shows the number of each type of configuration included in the survey. Most of the linacs in the survey were calibrated isocentrically to deliver 1 cGy/MU at 5 cm deep for a 10×10 cm field and deliver a nominal 740 MU/min. Modifications were made to the 740 MU/min plan for delivery on the 800 MU/min and 600 MU/min systems to ensure that MLC movements were consistent regardless of the dose‐rate. This was achieved by scaling the MU in the 740 MU/min plans by the ratio of dose rates. The 800 MU/min linac plans used more MU, and the 600 MU/min linacs used fewer. MU values in subsequent sections are those for the 740MU/min systems.

**TABLE 1:** Linac reference conditions.

1 cGy/MU depth (cm)	Dose rate (MU/min)	Number of linacs
1.4 (d_max_)	800	3
5	740	12
10	600	1

Participating centers were asked to perform dosimetry calibration prior to running the plans. This included updating the pixel correction map. Participating centres all followed Varian's preventative maintenance plan (PMP). Varian engineers service the linacs every 4 months. The variation in results between institutions is representative of the performance indices that should be expected under this regime.

Analysis code was developed for this project. It is free and open source.^7^ It was written in python and uses the matplotlib, numpy, pydicom and scipy libraries and their dependencies. The analysis was performed using commit August 14 2024 aa4d245be2, which uniquely identifies the version of the code used within the git repository.

The reported parameters are summarized in Table 2.

**TABLE 2:** Summary of plans and measured indices.

Plans (extra analysis on the same images)	Indices
Picket fence (leakage)	layer_max_dev
	layer_leakage_index
Sweeping gap (open field)	min_sweep, max_sweep, sweep_means_over_gantries, worst_prof_sweep_fit_perc, worst_asymmetry, worst_radial_asymmetry, output_variation_perc, beam_shape_change
Speed sweep	min_sweep, max_sweep, sweep_means_over_gantries, worst_prof_sweep_fit_perc
T2 GS	T2_GS_n_aaa
T2 DR	T2_DR_n_aaa
T3	T3_n_aaa

### 2.1 Picket fence and leakage

Apertures 1 mm wide were created centred at −130, −80, −30, 0 mm in one field and at 0, 30, 80, 130 mm in another. The fields were delivered at collimator angles of 90° and 270°. Using the images from the two collimator angles, the *Y* coordinate of the collimator rotation axis is found. This is taken as the origin of the leaf travel. The same technique for referencing the MLC positions to the collimator rotation axis has previously been used by Barnes et al.^8^ The position of the picket for each leaf pair is found. The overall largest absolute deviation from expected position over all collimator angles and picket positions is reported as the *layer_max_dev*.

The *layer_leakage_index* is the mean of half the image where there are no pickets. The image is intrinsically a ratio after having been divided by the open field. The open fields were 20 MU, and the picket fields were 80 MU, resulting in an index that is four times higher than the true leakage. As the DMI is not an energy‐independent detector, this index is only intended for comparison and change monitoring.

**FIGURE 1:**
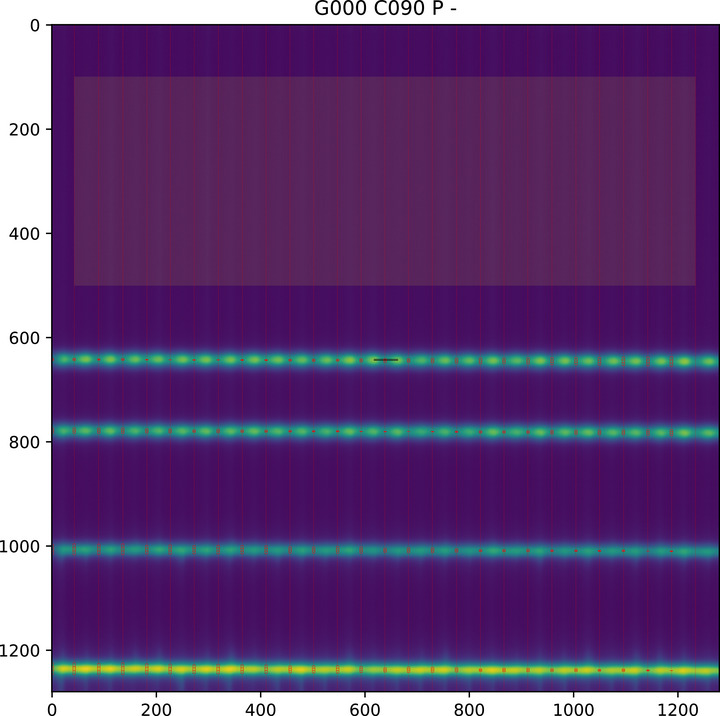
Example of automated picket fence analysis. The axes are labelled in pixels. The red dotted lines show where the profiles for each leaf pair have been taken using the lines of interleaf leakage to find each leaf. The short lines in the middle of the image show where the *Y* coordinate of the collimator rotation axis has been found using images acquired at collimator 90° and 270°. The lighter rectangular region shows where the layer leakage index is measured.

Figure 1 shows an example of the picket fence and leakage test.

### 2.2 Sweeping gap and open field

Chui et al. have used EPIDs to measure sweeping gaps.^9^ Rowshanfarzad et al. have used EPIDs to test individual leaf pairs.^10^

In this work, a 4 mm gap was chosen, swept in the positive and negative directions as recommended by the IPEM^11^ in report 81. This gap analysis is repeated at cardinal gantry angles to check for changes in performance with gravity. The open fields were 20 MU and the sweeping fields were 200 MU. The sweep images were divided by the open field images. This partially compensates for the FFF peak.

The interleaf leakage is found first, and the leaf‐pair profiles are taken between the interleaf leakage dose maxima. The mean is taken from each individual leaf pair profile. The mean for the whole image is calculated and the extreme values found over the images from each sweep direction and gantry angle (*min_sweep, max_sweep*). The mean is taken over images (*sweep_means_over_gantries*).

**FIGURE 2:**
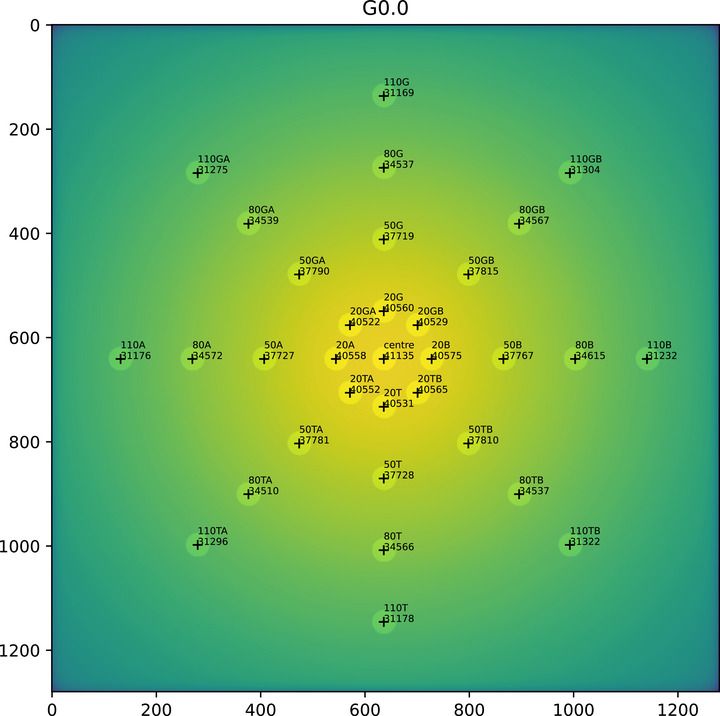
Open field analysis. The mean pixel value is found in circular regions. The labels describe the distance from the centre in millimetres and the direction, for example 80G is 80 mm in the direction of the gantry.

The open‐field delivery was repeated at each gantry angle. These images were analyzed for symmetry, changes in shape with gantry angle, and changes in output with gantry angle (Figure 2). The maximum percentage difference in the central region from gantry 0° is the *output_variation_perc*. The IPEM,^11^ report 81 recommend testing for output change with gantry angle every 3 months. The regions shown are analyzed and the indices *worst_asymmetry*, *worst_radial_asymmetry* are reported. Differences in the beam on opposite sides of the centre are described by *worst_asymmetry*. Asymmetry is not separately reported for inline and transverse directions. Instead, the asymmetry is calculated for these directions and diagonals and only the worst result is reported. The minimum to maximum difference in a ring of regions are described by *worst_radial_asymmetry*. Differences in the indices are expressed as a percentage of the field's central region. The regions are 10 mm circles with approximately 1600 pixels. The largest change in any region with gantry angle is reported as the *beam_shape_change*. The IPEM,^11^ report 81 recommend checking symmetry and flatness annually.

**FIGURE 3:**
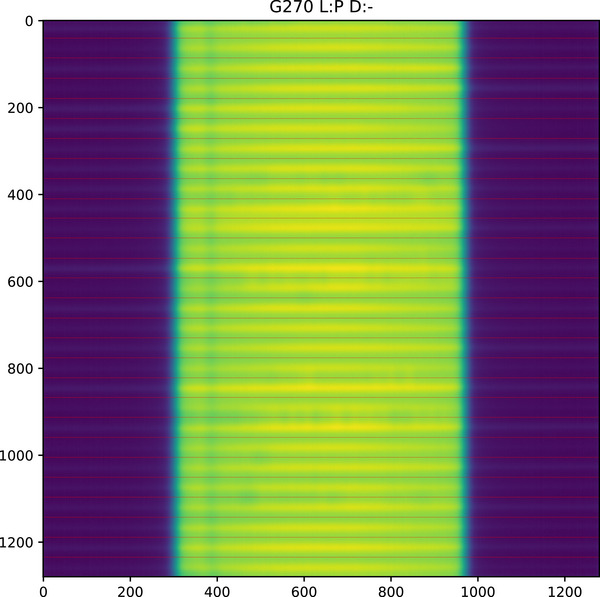
Sweeping gap image showing dose rate variation affecting all leaf pairs. Gantry 270, Layer: proximal, direction of leaf motion: negative.

**FIGURE 4:**
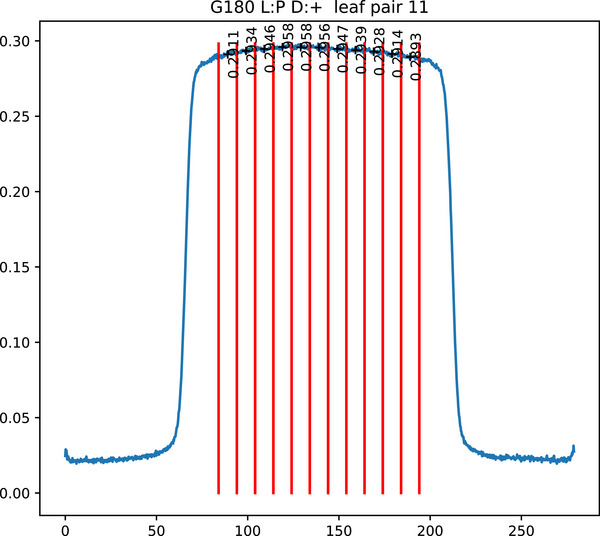
Analysis of a sweeping gap leaf pair profile.

Figure 3 shows an annotated image that has been automatically analyzed. Figure 4 shows a profile extracted from that image. The profile is taken from a five pixel wide strip, each point being the mean of five pixels to reduce noise. Each profile is split into 1 cm long sections. The maximum to minimum (range) within each profile is used as an index of sweeping gap consistency. Where a variation occurs in all leaf pair profiles, it indicates a variation in doserate, as in Figure 3.

**FIGURE 5:**
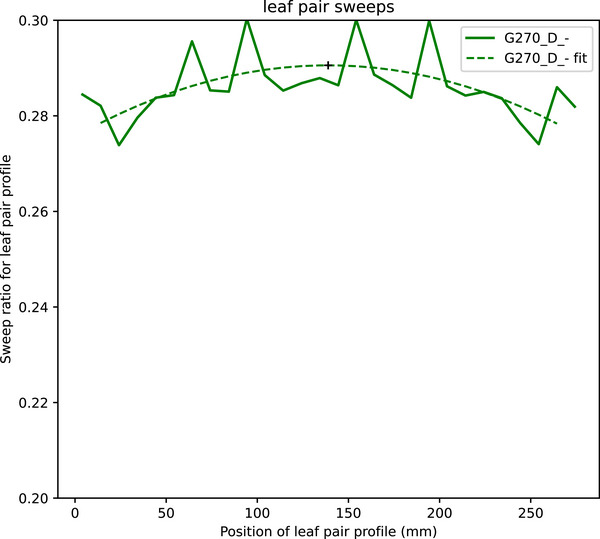
Leaf pair sweep values with quadratic fit.

Figure 5 shows that the sweep values vary across the field. The mean sweep for a leaf pair profile has been plotted against the distance from the edge of the field. The first step in the analysis is to divide the sweep image by the open field image to remove the FFF peak. It can be seen that this has not completely compensated. It is hypothesized that the slit aperture attenuates head scatter preferentially to focal photons from the target. The largest source of head scatter is likely to be the proximal aperture of the primary collimator. When a similar analysis is undertaken on a linac producing a flattened beam, the effect is more significant, as the flattening filter is a greater source of head scatter.

A quadratic is fitted to this curve. The largest magnitude percentage difference to the fit is reported as the worst_prof_sweep_fit_perc. Reporting the difference to a fit complicates the analysis but is beneficial because the variation from the curve is greater than the difference between leaf pairs in an image. The first and last leaf pair are excluded from the fit as the lack of lateral scatter results in those points being lower.

### 2.3 Speed sweep

Similar to the sweeping gap, a 4mm gap is used but with delivered monitor units reduced to 25. This results in MLCs moving a maximum speed across the field. The analysis is performed using the same method.

### 2.4 Ling T2 and T3

T3, T2 gantry speed (GS), and T2 dose rate (DR) following Ling^12^ were created. Unlike the other tests, the distal and proximal layers moved together for these test plans. For these tests, the images were not divided by open field images. For T2, the pixel regions were compared to the corresponding pixels in the open field. For T3, regions of pixels whose exposure was controlled by leaves moving at different rates where compared. The results are reported as signed percentage differences, for example *T2_GS_n_aaa*, where n in the number of the region and aaa is a 3‐digit gantry angle.

### 2.5 Plan specific QA

Hundred and three clinical plans were tested on two of the linacs at a single site. This had the benefit that consistent equipment could be used. An ArcCheck phantom^13^, ^14^ was used with a multiplug insert to hold a CC13 chamber^15^ at a point in the distribution where the dose gradient was as low as possible. The dose was calculated for the set up using Acuros Version 16.1.0 in the Eclipse (Varian) treatment planning system (TPS). The plans covered a range of SABR (lung, liver, pelvic node, and pancreas) and ICRU indications (lung, prostate and elective nodes, abdominal lymphoma, oesophagus, gynaecological, and rectum). Instead of a point reference dose, the mean dose to a structure representing the effective measuring volume of the chamber was used. This is particularly useful for SABR, where the dose can have a peak that is small compared to the detector. The dose distribution was also compared to TPS prediction using the ArcCheck's cylindrical diode array.

## 3 RESULTS AND DISCUSSION

### 3.1 Picket fence

**FIGURE 6:**
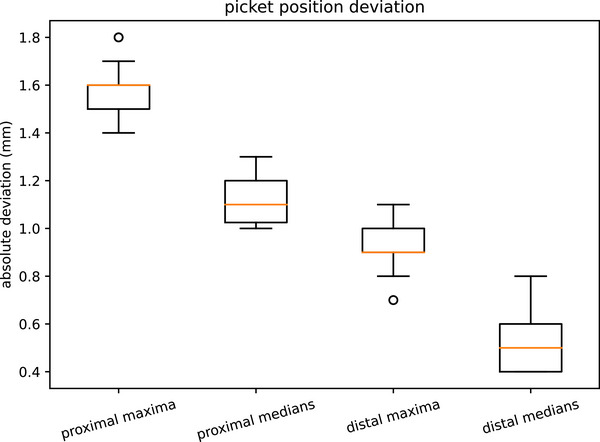
Picket fence box plot.

For each image, the median and maximum absolute positional deviation of each leaf pair was found. The median and maximum deviations found in the surveyed linacs are shown in Table 3 and Figure 6. The suggested tolerances are for individual leaf pairs. Every leaf pair of the distal layer met the TG142 recommendation^16^ of 1 mm in all positions apart from 1 linac. The first and last leaf pairs in the bank tend to have the highest deviations. Historically these may not have been tested.

**TABLE 3:** Picket fence results. Absolute difference from requested position.

	Proximal	Distal
Median	1.6 mm	0.9 mm
Maximum	1.8 mm	1.1 mm
Suggested warning tolerance	1.8 mm	0.9 mm
Suggested action tolerance	2.0 mm	1.0 mm

Rowshanfarzad et al. reported**10** the worst leaf deviation from the expected position for three linacs with Millenium 120 MLC over 5 months was 0.8mm. The single layer MLC is similar to the distal layer on the double layer MLC.

The layer leakage index (Table 4) is higher than the leakage ratio due to the ratio of MU between the open and picket fields. The DMI will also over‐respond to multiply scattered low‐energy photons present in greater proportion in the transmission acquisition.

**TABLE 4:** Layer_leakage_index.

	Median layer_leakage_index	Maximum layer_leakage_index	Suggested warning tolerance
Proximal	0.259	0.269	0.275
Distal	0.276	0.290	0.295

The linac that had the best picket fence result (worst individual leaf pair deviation in distal layer 0.7mm, worst in proximal layer 1.4mm) had not had any extra measures taken beyond the standard four monthly Varian service.

### 3.2 Sweeping gap and speed sweep

**FIGURE 7:**
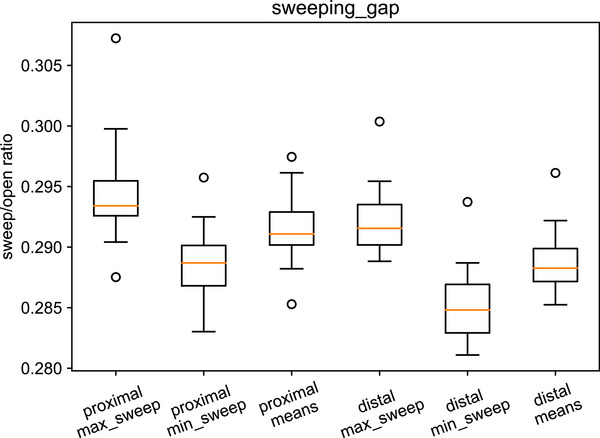
Sweeping gap box plot.

The sweeping gap results are summarized in Table 5 and Figure 7. The mean is taken over all the sweeps in an image. The maximum and minimum are taken over all gantry angles. The centiles of these are reported over the linacs in the study.

**TABLE 5:** Sweeping gap indices.

	Proximal		Distal	
max_sweep 75^th^ centile	0.296		0.294	
sweep_means_over_gantries mean	0.292		0.289	
min_sweep 25^th^ centile	0.287		0.282	
	Warning	Action	Warning	Action
Suggested max_sweep	0.300	0.310	0.300	0.310
Suggested min_sweep	0.280	0.270	0.280	0.270
Suggested mean	0.292 ± 0.005	±0.007	0.289 ± 0.005	±0.007

**FIGURE 8:**
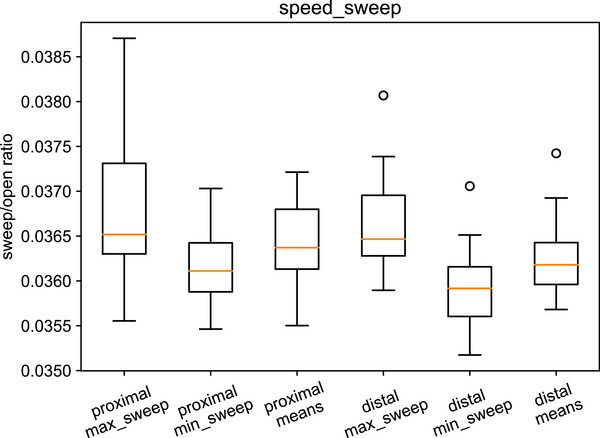
Speed sweep indices box plot.

The equivalent results are given for the speed sweep test in Table 6 and Figure 8.

**TABLE 6:** Speed sweep indices.

	Proximal		Distal	
max_sweep 75^th^ centile	0.0373		0.0370	
sweep_means_over_gantries mean	0.0365		0.0363	
min_sweep 25^th^ centile	0.0359		0.0356	
Suggested tolerance	Warning	Action	Warning	Action
max_sweep	0.0380	0.0390	0.0380	0.0390
min_sweep	0.0350	0.0340	0.0350	0.0340
mean	0.0365 ± 0.0005	±0.0007	0.0363 ± 0.0005	±0.0007

**FIGURE 9:**
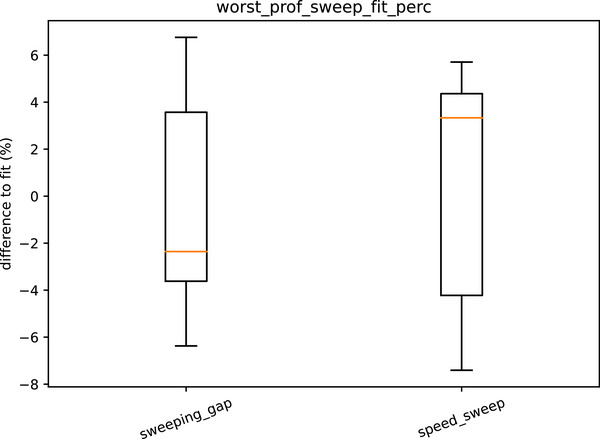
Box plot showing the variation of the sweeps within each sweep image.

Variation in performance of the leaf pairs within an image is given by the *worst_prof_sweep_fit_perc*. Results for this are shown in Figure 9. The suggested tolerances are in Table 7.

**TABLE 7:** Individual leaf pair sweep ratio difference to fit for proximal and distal layers.

Magnitude of difference to fit	Sweeping_gap	Speed_sweep
75^th^ centile	5.3%	5.3%
Maximum	6.8%	7.4%
Suggested warning	6%	6%
Suggested action	7%	8%

### 3.3 Open field analysis

**FIGURE 10:**
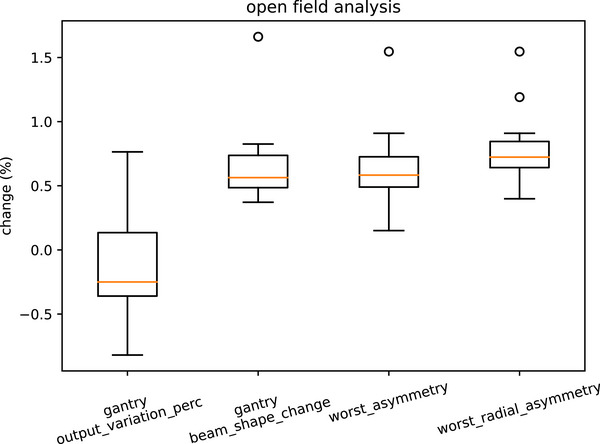
Box plot for indices from open field analysis.

The open‐field results are shown in Figure 10. Symmetry and changes in beam shape with gantry angle are less than 1%, apart from a few outliers. The output varies by less than 1% from the gantry 0 measurement. The suggested tolerances for the indices derived from the open fields are in Table 8.

**TABLE 8:** Open field indices suggested tolerances.

	Warning	Action
Output variation	0.8%	1.0%
Beam_shape_change	0.8%	1.0%
Worst_asymmetry	1.0%	1.5%
Worst_radial_asymmetry	1.0%	1.5%

### 3.4 Ling T2 GS, T2 DR, and T3

**FIGURE 11:**
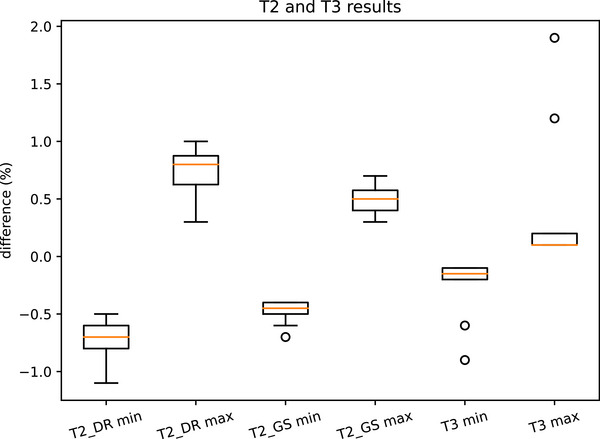
Box plot for T2 and T3 results.

It was found that all the Ling indices had an unbiased mean of 0.0%. The results and suggested tolerances are shown in Figure 11 and Table 9.

**TABLE 9:** Ling test percentage difference results.

	T2 GS		T2 DR		T3	
25^th^ centile of minima	−0.5%		−0.8%		−0.2%	
Mean	0.0%		0.0%		0.0%	
75^th^ centile of maxima	0.6%		0.9%		0.2%	
Suggested tolerance	Warning	Action	Warning	Action	Warning	Action
	0.8%	1.0%	1.0%	1.2%	0.8%	1.0%

Fogliata et al. reported^17^ five linacs had Ling T2 and T3 results within 2% for single layer Millenium MLC. These were mostly the older Clinac iX: it is expected that TrueBeam linacs would have a tighter spread as found here for Halcyon.

### 3.5 Plan specific QA

**FIGURE 12:**
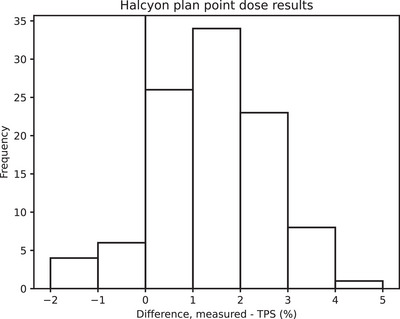
Histogram of results for clinical plans.

The point dose measurements were satisfactory with 69.9% of the measurements within 2% of TPS prediction (Figure 12). There is a systematic bias (mean 1.3%, two tail *p* value 5×10^−18^) in the difference, which requires further investigation. Lim et al. reported^2^ a similar discrepancy (median 1%) between TPS (AAA 15.6) and chamber measurement.

The relative gamma (3%, 2mm) pass rates were good, with 95 of 103 plans having pass rates higher than 98%.

## 4 CONCLUSIONS

A set of easily reproduced tests has been delivered and tested across 16 Varian Halcyon/Ethos linacs to establish a typical range of performance and tolerances. Plan specific QA has found that two of the linacs deliver plans precisely and accurately in line with clinical intent. This summary of results of repeated test plans can be used to give early warning that a machine may be performing outside the usual range for the class.

The proximal MLC layer did not meet the AAPM suggested tolerance of 1 mm for calibration accuracy. A double layer MLC had not been tried when the tolerance was suggested. We have found quality assurance results on clinical plans to be good. We suggest that because the collimation is shared between the MLC layers it is less significant if the one layer is more difficult to control. More work on the impact of the proximal layer calibration would be useful.

As discussed by Kim et al. Halycon 1.0 did not use the proximal layer for field shaping.**1** The optimizer created control points for the distal layer and the proximal layer stayed as close as possible, reducing interleaf leakage with minimal effect on the penumbra. With Halcyon 2.0 the proximal layer does shape the field. This has the advantage of allowing 0.5 cm steps in the field shape. As we have found the calibration of the proximal layer is not as good as the distal layer, it may mean that the deliveries are less accurate. Whether this is clinically significant requires further investigation.

## AUTHOR CONTRIBUTIONS

Andy Buckle: Test plan creation; EPI analysis code; project coordination; manuscript. Conor Heeney: Test plan editing; manuscript; plan delivery. Mathew Jones: Manuscript; plan delivery. Vasu Ganesan: Manuscript; plan delivery. Ronan Valentine: Manuscript; plan delivery. Philip Wheeler: Manuscript; plan delivery. James Earley: Manuscript; plan delivery. Matthew Sparks: Ling test plan creation; Ling test analysis code; manuscript. Garry Grogan: Test design discussion; manuscript.

## CONFLICT OF INTEREST STATEMENT

The authors declare no conflict of interest.
